# Pathology, Practical Medicine, and Therapeutics

**Published:** 1843-01

**Authors:** 


### PATHOLOGY, PRACTICAL MEDICINE, AND THERAPEUTICS.

### *On the Employment of large doses of Sulphate of Quinine in the treatment of Typhoid Fever.* By M. Saint Laurent.

Trials of the virtues of this remedy have been made at the Hôpital Cochin by M. Blache, who was led to form a conclusion favorable to its employment in cases of typhoid fever. The cases, however, in which it was used were not numerous, and in some of them other remedies were given either before or in connexion with the quinine. M. Broqua of Plaisance, who first introduced this practice, coming to Paris, induced M. Husson to permit some of his patients at the Hôpital Cochin, to be subjected to this mode of treatment, and the results thus obtained are published by M. Laurent.

The dose of the medicine was usually ten centigrammes every hour; sometimes the dose was larger and administered less frequently; and in several instances the patients took more than ʒij in every twenty-four hours for many days together. In thirteen cases no other remedy than the quinine was administered, but though the patients recovered, yet the results do not show any great superiority in this over other methods of treatment. In no instance were the symptoms cut short at once by the quinine, while in several cases the increased headach and thirst, and the greater dryness of the tongue which followed its use were not only of importance in themselves, but rendered the cases more complicated, since it was not easy to tell how far those symptoms were produced by the medicine, or how far they betokened an aggravation of the disease. Of ten patients who had the disease mildly, all recovered but one, whose death M. Laurent attributes, apparently with justice, to the action of the quinine. Of three patients who were attacked by the disease in a severe form, one only recovered, and even he was for some time in a state of great danger owing to hemorrhage from the intestines.

The cases are detailed in full, and are not by any means such as would impress one with a favorable opinion of the treatment proposed by M. Broqua. M. Laurent adds that M. Broqua is accustomed to administer the quinine in cases so slight that the patients would recover even though no treatment at all were adopted, and that if M. Husson had consented to its employment in such cases, the number of reputed cures from the sulphate of quinine would have been far greater. *Archives Générales. Sept.* 1842.

---

### *Report on the Results of Inoculation in Measles.* By Dr. M. Von Katona, of Borsoder, in Hungary.

In a very malignant and wide-spread epidemic of measles in the winter of 1841, the author inoculated 1122 persons with a drop of fluid from a vesicle, or with a drop of the tears of a patient with measles. The operation was performed in the same manner as the inoculation for smallpox. It failed in 7 per cent. of those on whom it was tried, but in all the rest it produced the disease in a very mild form, and not one of them died. At first a red areola formed round the puncture, but this soon disappeared: on the seventh day fever set in, with the usual prodromi of measles; on the ninth or tenth the eruption appeared; on the fourteenth desquamation commenced, with decrease of the fever and of the eruption; and by the seventeenth the patients were almost always perfectly well again. *Oesterreichische Medicinische Wochenschrift. Juli* 16, 1842.

---

### *Hairs growing on the Tongue.* By Dr. Beer, of Brunn.

The patient was a medical student, who after complaining for some time of dyspepsia and a *sticky* sensation in the mouth discovered hairs of considerable length grew from his tongue. They were detached in vomiting, but they grew again, and when the author saw him they were an inch long.

*Oesterreichische Medicinische Wochenschrift. August* 13, 1842.

*On the Chemical Composition of the Fluid of Ranula.* By L. Gmelin.

It is entirely different from saliva, containing no sulphocyanate, and only a small quantity of salivine, but chiefly albumen. The albumen, however, amounts to only 2 per cent. though the fluid is as thick as others containing about 5 per cent. It is to be supposed, therefore, either that the fluid of ranula contains some peculiar material to which it owes its thickness, or else that the albumen in it is of some peculiar kind.

*Repertorium für die ges. Medicin; from the Annalen der Chemie,* 1842, Hft. iii.

---

*The relative Frequency of Tubercles in various Organs.* By Dr. Engel, of Vienna.

The proportional frequency of the occurrence of tubercle in the lungs to that of tubercle in the cerebral membranes, the pleura, liver, and spleen, is as 18 to 1; to that of tubercle in the brain and kidney as 18 to 2; and that of tubercle in the peritoneum and intestines as 18 to 3. This is the more remarkable, when compared with the relative frequency of cancer in the same organs. Cancer of the lungs occurs, in proportion to cancer of the liver, as 18 to 48; to cancer of the stomach as 18 to 42; to cancer of the intestines and kidneys as 18 to 12; and to cancer of the brain, spleen, peritoneum and uterus, as 18 to 18. The frequency of tubercle of the lungs is to that of all other diseases of those organs as 2 to 3.

*Canstatt's Jahresbericht,* 1842.

---

*A new Method of administering Quinine.* By Dr. Guastamacchia.

The author's object was to find some method of avoiding the disgust which the bitterness of quinine always excites; and after repeated trials, he says he found it best to dissolve eight grains of the sulphate in half an ounce of rectified spirit and rub it, in two doses with an interval of a quarter of an hour between them, along the spine. In intermittent fever this should be done at the beginning of the cold fit; and it very often prevented even a single recurrence of it.

*Il Filiatre Sebezio. Agosto,* 1841.

---

*Account of an Epidemic of Cerebro-spinal Meningitis, observed at the Medical Clinque of the University of Strasburg.* By Professor Forget.

The description given by the Professor of this epidemic is so long, extending through several numbers of the Gazette Médicale, that we cannot afford space for more than the brief abstract of it contained in the Gazette des Hôpitaux.

The epidemic which began towards the close of 1840, and continued to prevail until May 1841, was almost exclusively confined to the poorer classes, and no cause could be assigned for its occurrence. Many writers have insisted on the suddenness of the attack, as one of the characteristics of the epidemic: there existed, however, a stage of premonitory symptoms, marked by occasional rigors, slight lassitude, headach, loss of appetite, &c. At the onset of the disease severe pain was felt in the forehead, temples, and occiput. This pain was sometimes constant, at other times it remitted at intervals; it was of a pulsating or lancinating character, or caused a sensation as though the head were bound with cords, or subjected to pressure, or as though it were being bored, or rent asunder. If the disease continued for any time the headach was succeeded by delirium or coma. It was accompanied by vertigo, confusion of the ideas, and hallucinations of the senses of sight or hearing. Pain in the back was a pathognomonic character of the epidemic. It was more frequently referred to the back of the neck than to the loins, and occasionally it was so severe that patients lay motionless on their back, not daring to move, for fear of exciting it afresh. Opisthotonos was often associated with the pain in the back, as was trismus, in some instances. The brows were knit, the eyes fixed, and risus sardonicus was often observed. There was generally considerable weakness and pain in the limbs; sometimes there was a condition of general or partial agitation, and subsultus tendinum was frequent in the advanced stages of the disease. In one instance there was a general trembling of the limbs, as in delirium tremens. In some cases there were clonic convulsions, and an epileptic seizure occurred in one instance. Delirium sometimes existed from the beginning, and coma without stupor accompanied or followed the delirium in some instances. Paralysis was rare. The eyes were affected in various ways: the conjunctiva was seldom injected, strabismus was rare, the pupil was sometimes dilated, sometimes contracted, occasionally natural. Optical illusions occurred sometimes, the sight was often obscured, but complete blindness was never noticed. Tinnitus aurium, deafness, and occasionally, deafness and loss of speech occurred in the same person. The face was occasionally turgid, but paleness of the face and integuments were more frequent, and burning heat of the skin was seldom noticed except of the forehead. As death approached the extremities became cold. An extensive eruption of *herpes labialis* was frequent, but did not appear to possess anything of a critical character. The tongue was usually moist and white at the beginning, and afterwards became dry, red, or brownish. As soon as the cerebral symptoms began to abate, the patients felt a desire for food. Thirst was usually not urgent. Vomiting at the commencement of the disease was of constant occurrence, and very severe. The bowels were generally constipated at first, afterwards purged. There was abdominal tenderness, either of the epigastrium, or of one side, usually the right, and as the disease advanced, the belly sometimes became distended and tympanitic. In many cases there was slowness of the pulse, which contrasted with the acute character of the other symptoms, and its beats were irregular. In some instances the bladder was paralysed.

*Course* of the disease, its *terminations*, its *forms*. In those who did not sink under the onset of the disease, the headach gave place to delirium, which was soon followed by coma. The vomiting ceased, but the pain and stiffness of the spine continued. Sometimes the disease remained stationary at this point so as to give rise to hopes of the patient's recovery. Gradual exhaustion, however, delirium at night, diarrhœa and obstinate vomiting came on, and ushered in the fatal termination.

*Post-mortem appearances.* The sinuses of the brain were gorged with blood. Sero-albuminous deposits on the parietal layer of the arachnoid, whenever fluid was contained in its cavity. A considerable quantity of sero-purulent fluid was found almost always in the lower part of the spinal canal. The pia mater was always injected, and this injection extended to the spinal marrow through a greater or less extent. On a level with the calamus scriptorius the arachnoid was frequently raised by a turbid serum effused beneath it. In the sub-arachnoid tissue were various deposits, sometimes resembling gelatin, at other times, like concrete albumen, or like true yellow pus, abounding especially about the optic nerves and around the origin of the spinal cord. Pus-globules were detected in this matter by the microscope. No important appearances were found in other organs, and in an anatomical point of view this epidemic presented nothing distinct from what is observed in sporadic meningitis, any more than it did in its symptoms.

*Treatment.* This consisted in free general depletion, repeated two, three, or four times in the course of two or three days, if the disease seemed to require it. Local depletion, either by leeches or cupping, produces, however, more marked relief than the general abstraction of blood. It appears, too, from the statistics furnished by M. Forget, that the patients who recovered lost more blood than those who died. The employment of calomel led to no good results whatever, but often produced obstinate diarrhœas, or affected the mouth severely. Tartar emetic, employed as a revulsive, did no good. M. Forget at length contented himself with mild laxatives to counteract the constipation. Blisters were frequently employed after or conjointly with depletion.

Febrifuge tonics, as quinine, antispasmodics, and excitants were tried and found to be injurious; but after the disease had been broken by antiphl ogistic measures, many of the nervous symptoms which remained were greatly relieved by the administration of opium.

*Gazette Médicale, Avril* 9-16-23, *Mai* 7-14, 1842; *and Gazette es Hôpitaux, Juin* 2, 1842.

---

*Account of a Disease which affected a large number of Persons in the Canton Zurich, and which was induced by partaking of Decayed Meat.* By Dr. Sigg.

On June 10th, 1839, a musical festival was held in the church at Andelfingen, to which persons resorted from all parts of the canton. In the church the air was extremely hot, and the atmosphere out of doors was oppressive, and the clouds were heavy, and threatened a thunder storm. After spending four hours in the church, between 500 and 600 persons sat down in an outhouse, which had been fitted up for the occasion, to a dinner of cold veal, ham, and sallad, with indifferent wine and beer. The meat did not look good, and the ham had such a strong taste that many persons did not partake of it, though most made a hearty meal.

On their way home many were taken ill and vomited; on the following day many more were attacked with nausea, vomiting, and diarrhea, and within a week or ten days, most who had borne a part in the festival became more or less seriously indisposed. Of the 600 persons who had partaken of the dinner, 444, or more than two thirds were attacked between the 10th and 20th of June, of whom nine died with typhoid symptoms.

Those persons who had vomited on their way home from the festival, suffered but little subsequent inconvenience; the others were mostly seized from the 5th to the 10th day after it, with sense of uneasiness and exhaustion, with pains in the limbs and head, slight rigor, loss of appetite, with great thirst, and an extremely unpleasant coppery taste in the mouth. Nausea, with vomiting and diarrhea, were extremely common, and notwithstanding their sense of exhaustion, the patients were unable to sleep, but walked listlessly about, or threw themselves on the ground to rest.

After these symptoms had lasted about seven days, they increased in severity; the fever ran higher, the pains in the head were more distressing, and the patients were compelled to keep their bed. Colic-pains came on in the abdomen, and the epigastric region was frequently extremely tender. At this stage of the disease constipation usually existed, but sometimes diarrhea came on, in which the patients passed six or eight dark-green and highly-offensive evacuations in the twenty-four hours, by which they were greatly exhausted. Delirium often accompanied the evening exacerbations of fever, during which the patients were extremely restless, and often violent, but about the 12th day they usually sank into a torpid state with predominance of the typhoid symptoms while it was only on touching the abdomen that they seemed to be suffering pain. Symptoms of oppression at the chest generally supervened from the 14th to the 17th day, and were attended with cough and slight expectoration. This occurrence seemed to mark the third stage of the disease, and was usually accompanied with the gradual subsidence of the abdominal symptoms. Moisture appeared at the edges of the tongue, the skin began to perspire, and the patients gradually advanced towards convalescence. No discharge occurred which could be called critical, but the cough and fever usually subsided together, leaving the patient in a state of great weakness. In the cases which terminated fatally, the typhoid symptoms of the second stage increased in severity, the abdomen became tympanitic, the stools were horribly offensive, and often mixed with blood, the weakness of the patient became extreme, and fainting-fits were frequent, till death at length took place.

A post-mortem examination was made in four cases. The membranes of the brain were found congested; and a considerable quantity of bloody serum escaped from the cut surface of the lungs. The heart was flabby, but its muscular substance did not appear altered. The most important changes were found in the lower part of the ileum, which was red externally; and on laying it open the mucous membrane was found of a dark-red colour in patches, very friable, or if the disease were further advanced, presenting patches of ulceration varying in size from that of a mustard seed to a fourpenny-piece.

The writer enters into a long examination of the causes of the disease, far too long to admit of condensation here. It appears, however, to be clearly referrible to the meat of which the persons partook, none having suffered who ate only bread and cheese, or butter, while persons who were not present at the festival, but who partook of the meat at their own homes were affected. The animals appear to have been healthy, and their flesh seems to have been perfectly good when they were first killed, but to have become tainted by being kept for some days, after it was cooked, in an ill-ventilated cellar, while the hams were originally but imperfectly cured. *Hufeland's Journal. May*, 1841.

---

*Quinine in Asthma.* By B. R. HOGAN, Esq. United States.

THE author asserts that quinine, administered in doses of from two to eight grains, repeated in an hour, if relief does not follow, has cured every case of asthma in which he has tried it. He was induced to try it in asthma, in consequence of its known efficacy in all paroxysmal and congestive diseases. He also alleges that in the "forming stage" of croup, in the case of a child, two years old, a couple of grains of quinine, and a "snuff" plaster on the chest, warded off the attack. *American Medical Intelligencer. No.* viii. *Feb.* 1842.

